# Heterozygous pathogenic variants in *SERPINB7* potentially associated with Nagashima-type palmoplantar keratoderma and Hashimoto’s thyroiditis: a case report

**DOI:** 10.1515/biol-2025-1299

**Published:** 2026-05-25

**Authors:** Hong Liang, Yuwen Chen

**Affiliations:** Department of Pediatrics, Hainan General Hospital (Hainan Affiliated Hospital of Hainan Medical University), Haikou, Hainan, 570100, China

**Keywords:** Nagashima-type palmoplantar keratoderma, Hashimoto’s thyroiditis, serpin family B member 7, immune dysregulation

## Abstract

Nagashima-type palmoplantar keratoderma (NPPK) is an autosomal recessive disorder caused by biallelic loss-of-function variants in the *SERPINB7* gene, whereas Hashimoto’s thyroiditis (HT) is a common autoimmune endocrine disease. The co-occurrence of NPPK and HT has not been previously reported. This study aims to present this novel association and investigate a potential underlying immunogenetic mechanism. We describe a 10-year-old boy presenting with fever, seizures and a long-standing history of palmoplantar erythema and hyperkeratosis. Diagnostic work-up included neurological evaluation, thyroid function tests, cytokine profiling and whole exome sequencing (WES). Laboratory findings during the acute phase revealed elevated anti-thyroid antibodies (anti-thyroid peroxidase antibodies, anti-thyroglobulin antibodies) and pro-inflammatory cytokines (interleukin [IL]-6, IL-10, interferon-γ). Skin examination confirmed NPPK, and WES identified compound heterozygous pathogenic *SERPINB7* mutations (c.522dupT; p.V175Cfs*46). The patient was diagnosed with NPPK, HT and Hashimoto’s encephalopathy. Treatment with levothyroxine and levetiracetam led to the resolution of neurological symptoms and normalisation of thyroid function. This first report of coexisting NPPK and HT suggests a potential novel role for *SERPINB7* in immune dysregulation beyond its known cutaneous functions. The findings advocate considering thyroid screening in patients with NPPK and expand the phenotypic spectrum of *SERPINB7*-related disorders, warranting further research into its extracutaneous immunological roles.

## 1 Introduction

Nagashima-type palmoplantar keratoderma (NPPK) is a rare autosomal recessive hereditary skin disorder characterised by well-demarcated erythema, hyperkeratosis and hyperhidrosis of the palms and soles, with lesions potentially extending to the dorsal hands, feet, wrists and ankles. This disorder is caused by biallelic pathogenic variants in the *SERPINB7* gene, which encodes a member of the serine protease inhibitor (serpin) superfamily, proteins known to regulate proteolytic activity and maintain epithelial barrier function and immune homeostasis [1], [2], [3]. The disorder shows a relatively high prevalence in East Asian populations, and numerous pathogenic mutations in *SERPINB7* have been identified, including recurrent and novel variants [4]. Although the clinical diagnosis of NPPK is typically confirmed by genetic testing [5], 6], ongoing research continues to elucidate its pathomechanism, with recent studies exploring deep intronic variants and splicing dysregulation [7].

Hashimoto’s thyroiditis (HT) is a common autoimmune thyroid disorder characterised by chronic lymphocytic infiltration and progressive destruction of the thyroid gland, leading to hypothyroidism [8]. Although more prevalent in middle-aged adults, it can occur in paediatric patients [9]. The pathogenesis of HT involves a complex interplay of genetic predisposition, environmental triggers and immune dysregulation, particularly involving autoreactive T cells and the production of anti-thyroid antibodies such as anti-thyroid peroxidase antibodies (TPOAb) and anti-thyroglobulin antibodies (TgAb) [10], 11].

The potential immunological role of *SERPINB7* extends beyond skin barrier integrity. Evidence suggests that *SERPINB7* is expressed not only in keratinocytes but also in lymphoid tissues [12]. Its deficiency has been linked to dysregulated immune responses and has been shown to exacerbate skin inflammation and upregulate inflammatory cytokines such as tumour necrosis factor-α, interleukin (IL)-1β, IL-23 and interferon (IFN)-γ in psoriasis models [13], 14], indicating its involvement in inflammatory and autoimmune pathways. Furthermore, T lymphocytes have been implicated in the pathogenesis of NPPK, suggesting a shared immunological basis that may extend to other autoimmune conditions [15], 16]. Although *SERPINB7* variants have not been widely reported in association with extracutaneous autoimmune diseases, the gene’s role in immune homeostasis warrants further exploration.

Other genetic variants have been independently associated with either NPPK or autoimmune thyroid diseases (AITDs) [17], 18], but no overlapping genetic predisposition has been firmly established between NPPK and HT. Genes involved in immune regulation and skin integrity, such as those encoding proteins in the serpin superfamily or human leukocyte antigen (HLA) regions, may represent potential common pathways [19], though population-specific predispositions remain underexplored.

This case report aims to investigate the hypothesis that heterozygous pathogenic variants in *SERPINB7* may contribute to the co-occurrence of NPPK and HT through mechanisms of immune dysregulation. To explore this, we employed WES for genetic analysis, alongside cytokine profiling and thyroid autoantibody assays, to delineate the immunogenetic profile of the patient. By integrating clinical, genetic and immunological data, this study seeks to expand the phenotypic spectrum of *SERPINB7*-related disorders and critically evaluate its potential role in autoimmune regulation, thereby addressing a gap in the current literature regarding shared genetic susceptibility in dermatological and endocrine autoimmunity.

## 2 Case presentation

### 2.1 Data collection

The patient in this case report was identified and clinically evaluated at the Neurological Institute of the Department of Paediatrics, Hainan General Hospital. Written informed consent for clinical care and genetic testing was obtained from the participant’s adoptive parents and approved under Hainan General Hospital’s protocol. The signed patient consent form for participation in this case report, including the use of de-identified photographic images, was obtained from both the participant and their legally authorised representative.

**Informed consent:** Informed consent has been obtained from all individuals included in this study.

**Ethical approval:** The research related to human use has been complied with all the relevant national regulations, institutional policies and in accordance with the tenets of the Helsinki Declaration, and has been approved by the Ethics Committee of Hainan General Hospital (Yi Lun Yan [2025] No. 314).

### 2.2 Patient profile

On 24 April 2025, a 10-year-old boy was admitted to Hainan General Hospital due to ‘fever for 4 days, with intermittent convulsions for over 3 h’. The patient had a history of asymptomatic erythema and thickening of the palms and soles beginning at age 3. Before admission, oseltamivir was taken orally for 3 days, and 5 mg of diazepam was administered to control convulsions.

### 2.3 Chief complaints and admission history

The patient mainly presented with fever, with the highest body temperature reaching 41 °C. Four days later, he lost consciousness and fell to the ground; his eyes were fixed, his teeth were clenched and he experienced limb convulsions lasting approximately 30 s. Subsequently, his condition improved, with no vomiting or incontinence. His condition remained stable without progression, and he sought further diagnosis and treatment at our hospital.

### 2.4 Admission examination

**Figure 1:**
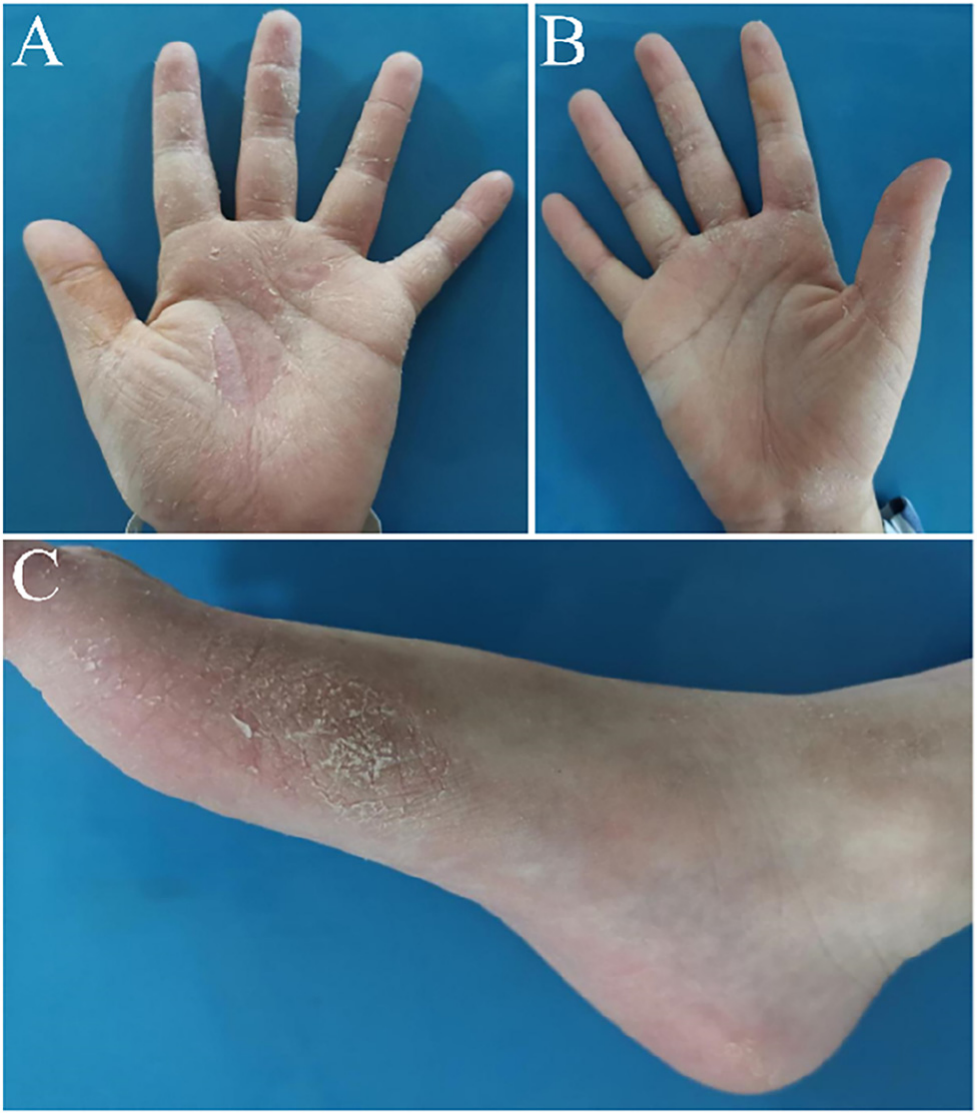
Clinical features of the proband. Images revealing bilateral symmetrical, well-demarcated diffuse erythema and hyperkeratosis over the palms and soles. A. left hand, B. right hand, C. right foot.

On admission, his vital signs were stable. Neurologically, his Glasgow Coma Scale (GCS) score was 6/15. Physical examination revealed bilateral, symmetrical palmar and plantar hyperkeratosis extending beyond the volar margins, accompanied by hyperhidrosis, tinea pedis and foot odour (Figure 1). Tremors were observed in both upper limbs.

### 2.5 Laboratory tests

**Figure 2:**
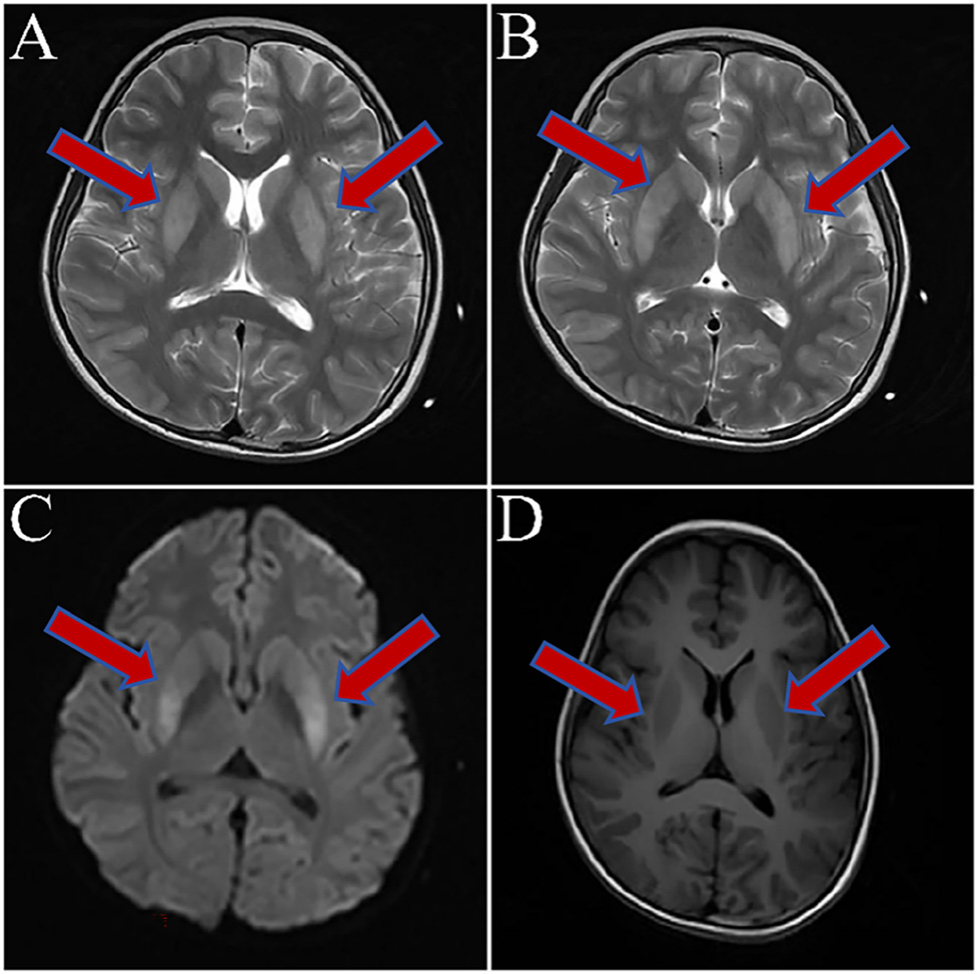
MRI showed symmetrical abnormal signals in the bilateral basal ganglia region. A–D demonstrated a nodular high signal in the both sides of the basal ganglia (white arrows) on T2WI (A), FLAIR (B), and DWI (C) while presented low signal on T1WI (D).

**Figure 3:**
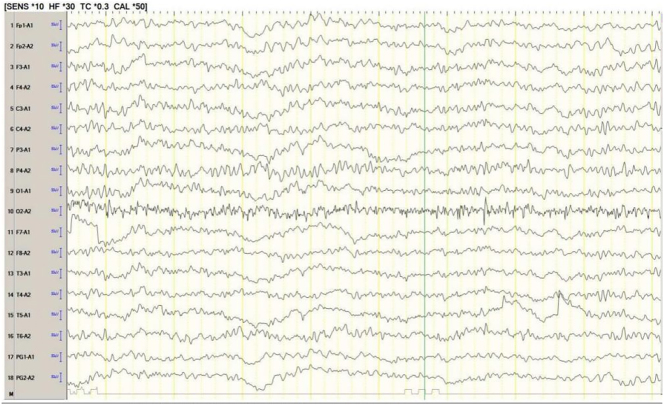
The EEG of the patient was tested upon admission. It showed paroxysmal medium to high amplitude 4–5 Hz theta waves, and a few 1.5–3 Hz.

Serial laboratory monitoring was conducted to assess disease progression and treatment response. The following initial laboratory investigations were performed during the stable period after admission:

– **Thyroid function**: Thyroid-stimulating hormone (TSH) at 0.3314 mIU/L, free triiodothyronine (FT3) at 2.34 pmol/L, free thyroxine (FT4) at 12.89 pmol/L, TPOAb at 39.07 IU/mL and TgAb at 38.68 IU/mL.
– **Cytokine levels**: Interleukin-6 at 10.05 pg/mL, IL-10 at 8.86 pg/mL and IFN-γ at 22.34 pg/mL.
– **Magnetic resonance imaging (MRI)**: Small patchy foci of prolonged T1 and T2 signals were observed on both sides of the basal ganglia, with slightly higher signals on fluid-attenuated inversion recovery and diffusion-weighted imaging (Figure 2).
– **Electroencephalography (EEG)**: Paroxysmal medium-to-high amplitude 4–5 Hz theta waves, with a few 1.5–3 Hz delta waves in the left cerebral hemisphere (Figure 3).
– **Cerebrospinal fluid (CSF)**: The CSF was colourless and clear, with a leukocyte count of 2/mm^3^; intracranial pressure was 170 mm H_2_O. Cytology was lymphocytic, and CSF biochemistry suggested normal protein, sugar and chloride levels. The results of metagenomic next-generation sequencing of the CSF were negative.
– **Other**: A specific neuronal autoantibody panel (anti-NMDAR, VGKC-complex, and GAD65) was performed and returned negative results. No major abnormalities were detected in full blood count, rheumatoid factor, antisense oligonucleotides (ASOs), antinuclear antibody test, antineutrophil antibodies, lupus anticoagulant, infectious disease screening, electrolytes, liver and renal function, glycolipid biochemistry, folic acid and vitamins, tumour markers and T-SPOT.
– **Notes:** It is noteworthy that comprehensive immune profiling, including Th17 and regulatory T-cell markers, was not feasible due to limitations in available laboratory assays at the institution. The clinical presentation – characterised by acute encephalopathy in the context of newly identified thyroid autoimmunity, typical EEG findings for encephalopathy and the absence of specific features suggestive of limbic encephalitis or other classic autoimmune encephalitides – led to a primary diagnosis of Hashimoto’s encephalopathy (HE), guiding the initial diagnostic and therapeutic priorities.

**Figure 4:**
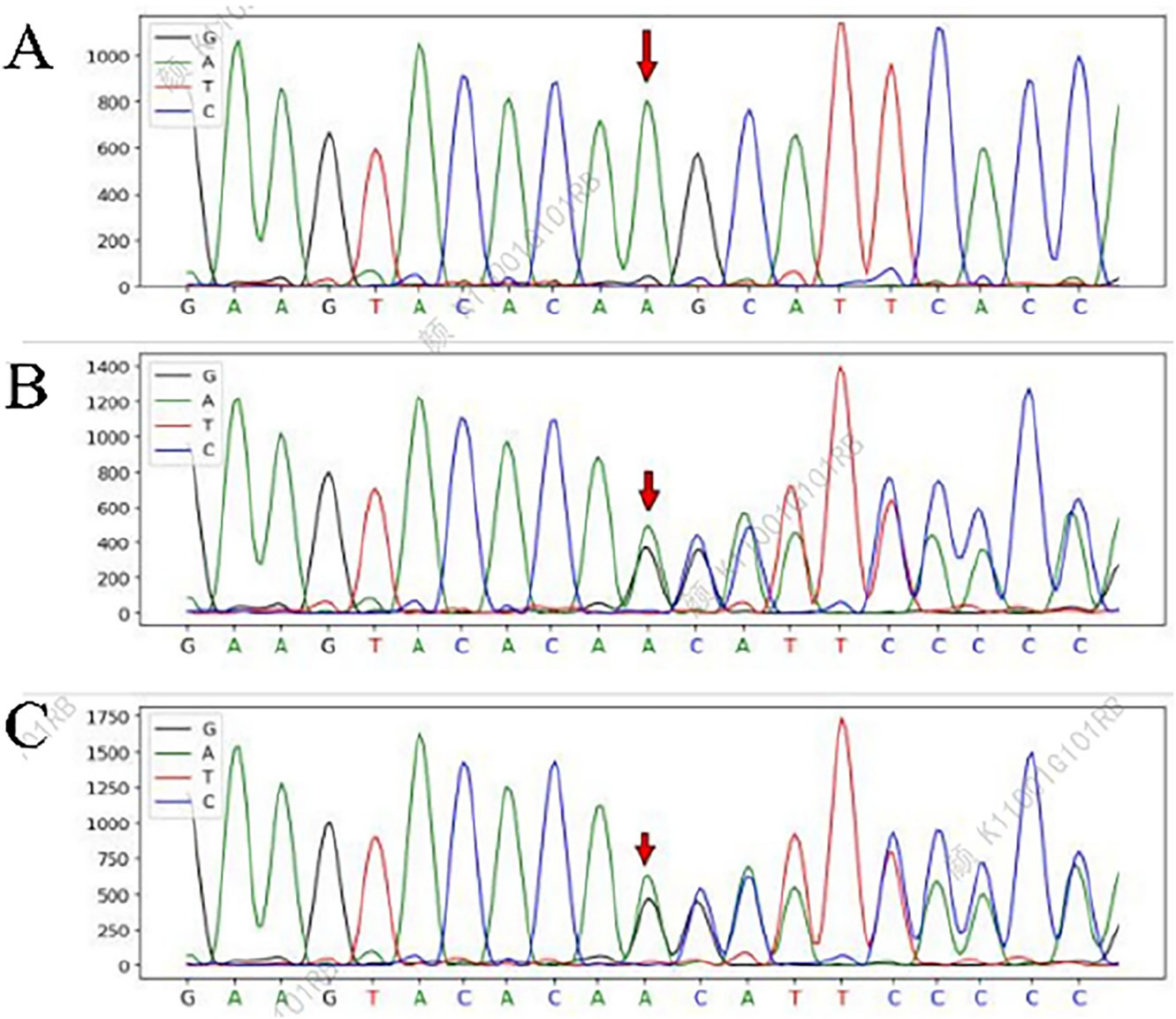
Whole exome sequencing of the patient and his immediate family members (parents). (A) The heterozygous c.522dupT (p.V175Cfs*46) variant in the SERPINB7 gene identified in the patient; (B) the patient’s father’s SERPINB7 gene; (C) the patient’s mother’s SERPINB7 gene.

### 2.6 Genetic analysis

– Whole exome sequencing revealed compound heterozygous variants in the *SERPINB7* gene: c.522dupT (p.V175Cfs*46) (Figure 4). In silico bioinformatic analysis using REVEL (Pearson Education, Inc, Hoboken, New Jersey 07030, US) and SpliceAI (Illumina, Inc, San Diego, California, USA) was performed to predict the functional impact of the identified variants, supporting a deleterious effect.

### 2.7 Key diagnoses

– **Hashimoto’s thyroiditis**: Thyroid function test results were consistent with HT [20], including elevated circulating concentrations of autoantibodies (particularly TPOAb and TgAb), altered TSH levels and corresponding clinical and biochemical features [21].
– **Nagashima-type palmoplantar keratoderma**: Skin examination showed symmetrical, well-demarcated diffuse erythema and hyperkeratosis over the palms and soles, accompanied by hyperhidrosis of the same regions.
– **Genetic findings**: Genetic analysis detected a *SERPINB7* gene variant, c.522dupT (p.V175Cfs*46).
– **Hashimoto’s encephalitis**: Central nervous system infections, space-occupying lesions and autoimmune encephalitis were excluded. Based on the symptoms (myoclonus and seizures) and the presence of positive TPOAb and TgAb, a diagnosis of HE was made. Thyroid function tests, MRI examination and EEG monitoring were consistent with this diagnosis.

### 2.8 Treatment course

The patient was initially treated in the hospital with intravenous levetiracetam (500 mg) for 24 h, followed by an oral regimen starting at 250 mg twice daily. The dose was increased to 500 mg twice daily after 2 weeks and further increased to 750 mg twice daily after an additional 2 weeks.

### 2.9 Outcome

The patient demonstrated marked clinical improvement following the initiation of treatment. The seizures ceased, and his neurological status recovered fully, with the GCS score improving to 15/15 by day 10 of hospitalisation. A repeat electroencephalogram performed before discharge showed normalisation of previous abnormalities. The transient tremors in the upper limbs resolved, and the patient was discharged after 14 days of inpatient care.

At the 4-week outpatient follow-up, the patient remained seizure-free without recurrence of neurological symptoms. Thyroid function tests showed TSH at 1.056 mIU/L (reference: 0.60–4.40), FT3 at 3.4 pmol/L (3.45–5.50) and FT4 at 11.36 pmol/L (11.00–17.70), with a notably elevated TPOAb of 78.04 IU/mL (<5.61). He was advised to continue the current levetiracetam regimen and was monitored off levothyroxine, reflecting the transient nature of his thyroid dysfunction in the context of the acute illness. The dermatological findings of palmoplantar keratoderma persisted, consistent with the chronic genetic nature of NPPK.

Upon re-evaluation on 8 May 2025 during the recovery phase and subsequent testing on 9 September 2025 during a stable period, thyroid function and autoantibodies (TPOAb and TgAb) were found to have normalised. Furthermore, after levothyroxine was discontinued for 1 month, a follow-up assessment on 6 October 2025 confirmed that thyroid function and antibody levels remained within normal limits.

## 3 Discussion

Recent functional studies on the SerpinB7 protein, encoded by *SERPINB7*, highlight its critical role in keratinocyte differentiation and skin barrier integrity. SerpinB7 deficiency has been linked to disrupted epidermal barrier function, increased keratinocyte proliferation and altered inflammatory responses, contributing to skin conditions such as psoriasis and NPPK [22]. The identified variant in our patient, c.522dupT (p.V175Cfs*46), is a frameshift mutation predicted to result in a truncated protein, and its classification as pathogenic is supported by ACMG guidelines due to its null nature (predicted loss of function) and its presence in trans with another mutant allele in a recessive disorder [23]. Although NPPK typically requires biallelic loss-of-function variants, the potential implications of heterozygosity, particularly in the context of immune dysregulation, warrant consideration. Although the literature directly supporting pathogenicity from a single allele in NPPK is limited, haploinsufficiency or a dominant-negative effect in specific cellular contexts, particularly within immune pathways, cannot be entirely excluded and may be influenced by modifier genes or other genetic factors.

Hashimoto’s thyroiditis is one of the most common AITDs, in which the immune system attacks the thyroid gland, leading to chronic inflammation and, eventually, thyroid dysfunction [24], 25]. This condition can manifest in diverse ways, ranging from subtle to life-threatening. Although the typical presentation involves fatigue, weight gain, sensitivity to cold, dry skin, hair loss, muscle weakness and depression, some exceptional presentations include HE, which mimics seizures in less than 1 % of patients [26], 27]. The diagnosis of HT is primarily based on the clinical symptoms of hypothyroidism, the presence of TPOAb and TgAb and thyroid ultrasound. Approximately 60–80 % of patients with HT are TgAb positive, and up to 95 % are TPOAb positive [28]. The immune response in HT is driven by both cellular and humoral mechanisms, with autoreactive T cells playing a pivotal role in its pathogenesis [29]. These autoreactive T cells target thyroid antigens, leading to inflammation and gradual loss of thyroid function [30], 31].

Although *SERPINB7* mutations are firmly linked to NPPK, their role in immune dysregulation is an emerging concept. Published evidence suggests that *SERPINB7* deficiency can exacerbate skin inflammation and alter cytokine expression in models such as psoriasis [22]. Furthermore, a lead SNP in the *SERPINB7* intron (rs12964116) has been shown to affect transcription factor binding sites for STAT3 and C/EBPβ, which are critical for immune cell maturation and Th2 cytokine regulation [32]. This supports a potential role for *SERPINB7* in immune pathways beyond its function in keratinocytes. The immunogenetic link between *SERPINB7* and HT proposed here remains speculative and hypothesis generating. A potential mechanism could involve *SERPINB7* dysfunction impairing immune tolerance in the skin and, potentially, systemically through altered processing of antigens or dysregulation of protease activity crucial for T-cell receptor signalling or cytokine activation [33]. This could lower the threshold for autoimmunity, potentially in conjunction with other genetic factors such as specific HLA haplotypes known to confer risk for AITDs [34]. The neurological presentation of HE in our case, though not directly mechanistically linked to *SERPINB7* in the literature, could be indirectly mediated by the systemic inflammatory milieu and autoantibody production driven by the underlying immune dysregulation. The co-occurrence of NPPK and HT in this patient, though previously unreported, may suggest a shared underlying immunogenetic predisposition mediated by *SERPINB7* dysfunction rather than a mere coincidence.

In this case, the expression levels of IL-6, IL-10 and IFN-γ in the peripheral blood of the proband were increased. As such, NPPK may be linked to HT through immune dysregulation mediated by *SERPINB7*-associated protein abnormalities. The clinical implication of our findings, if validated in larger cohorts, would be the potential benefit of screening patients with NPPK for thyroid dysfunction and autoantibodies. This could allow for earlier detection and management of associated autoimmune conditions. Therapeutically, our case opens a discussion on potential targeted interventions. If a specific inflammatory pathway, such as the IL-23/Th17 axis or the JAK/STAT pathway activated by cytokines such as IFN-γ, is confirmed to be involved, then biologics or JAK inhibitors used in other autoimmune diseases could be considered [35]. Furthermore, the recent identification of deep intronic variants in *SERPINB7* causing NPPK via splicing defects suggests ASOs as a future therapeutic strategy for the genetic defect itself [7].

This study has several limitations. The single-case nature precludes definitive conclusions about causality. Segregation analysis within the family was not possible in this case, which is a recognised limitation. The absence of familial genetic data limits understanding of the inheritance and penetrance of the phenotype. The immune profiling was incomplete, lacking data on Th17 cells, regulatory T cells and a broader cytokine panel. Furthermore, we could not perform functional assays to validate the impact of the identified *SERPINB7* variant on protein function or immune cell activity. Although familial genetic testing was unavailable to further clarify inheritance patterns and penetrance, the finding prompts a re-evaluation of whether subtle thyroid or autoimmune features might have been overlooked in previous NPPK cohorts. A comparison with the existing literature on NPPK indicates that systematic screening for thyroid autoimmunity has not been routinely reported, highlighting both a potential gap and the novelty of our observation.

Future directions should include functional studies to elucidate the precise role of *SERPINB7* in immune cells and tolerance pathways. Genetic screening of larger NPPK cohorts for thyroid autoimmunity and *SERPINB7* variants, including assessment of heterozygotes, is necessary to establish prevalence and genotype–phenotype correlations. Exploration of established autoimmune susceptibility genes, such as HLA, in patients with both NPPK and HT could identify potential genetic interactions. Ultimately, such research will clarify whether the association observed here is causal or coincidental and inform potential screening protocols and targeted therapeutic strategies.

## 4 Conclusions

This case report describes the first documented co-occurrence of NPPK and HT in a paediatric patient with compound heterozygous pathogenic variants in the *SERPINB7* gene, suggesting that abnormalities in *SERPINB7*, a gene traditionally associated with skin barrier integrity, may contribute to broader systemic immune dysregulation, potentially predisposing individuals to autoimmune conditions such as HT. This expands the known phenotypic spectrum of *SERPINB7*-related disorders beyond the skin. From a clinical perspective, these observations suggest that patients presenting with NPPK may benefit from screening for thyroid autoimmunity. For the research community, this case underscores the need to further investigate the extracutaneous immunological roles of *SERPINB7*, using functional assays and larger cohort studies to validate this potential association and elucidate the underlying mechanisms.

## References

[1] Kubo A, Shiohama A, Sasaki T, Nakabayashi K, Kawasaki H, Atsugi T (2013). Mutations in *SERPINB7*, encoding a member of the serine protease inhibitor superfamily, cause Nagashima-type palmoplantar keratosis. Am J Hum Genet.

[2] Kelly-Robinson GA, Reihill JA, Lundy FT, McGarvey LP, Lockhart JC, Litherland GJ (2021). The serpin superfamily and their role in the regulation and dysfunction of serine protease activity in COPD and other chronic lung diseases. Int J Mol Sci.

[3] Liu J, Chen Z, Hu L, Song Z, Mo R, Tsang LS (2023). Investigation of Nagashima-type palmoplantar keratoderma in China: a cross-sectional study of 234 patients. J Dermatol.

[4] Xiao T, Liu Y, Wang T, Ren J, Xia Y, Wang X (2022). Two novel mutations of *SERPINB7* in eight cases of Nagashima-type palmoplantar keratosis in the Chinese population. J Dermatol.

[5] Hannula-Jouppi K, Harjama L, Einarsdottir E, Elomaa O, Kettunen K, Saarela J (2020). Nagashima-type palmoplantar keratosis in Finland caused by a SERPINB7 founder mutation. J Am Acad Dermatol.

[6] Huang C, Yang Y, Huang X, Zhou Z (2021). Nagashima-type palmoplantar keratosis: clinical characteristics, genetic characterization, and clinical management. Biomed Res Int.

[7] Chen Y, Chen Z, Li S, Liu J, Liu Y, Fu Q (2025). A deep intronic founder variant in the *SERPINB7* gene causing aberrant splicing is a potential therapeutic target for Nagashima-type palmoplantar keratoderma. J Dermatol Sci.

[8] Kaur J, Jialal I (2025). Hashimoto thyroiditis. StatPearls [Internet].

[9] Hu X, Chen Y, Shen Y, Tian R, Sheng Y, Que H (2022). Global prevalence and epidemiological trends of Hashimoto’s thyroiditis in adults: a systematic review and meta-analysis. Front Public Health.

[10] Ferrari SM, Fallahi P, Elia G, Ragusa F, Ruffilli I, Paparo SR (2020). Thyroid autoimmune disorders and cancer. Semin Cancer Biol.

[11] Ragusa F, Fallahi P, Elia G, Gonnella D, Paparo SR, Giusti C (2019). Hashimotos’ thyroiditis: epidemiology, pathogenesis, clinic and therapy. Best Pract Res Clin Endocrinol Metab.

[12] Uhlén M, Fagerberg L, Hallström BM, Lindskog C, Oksvold P, Mardinoglu A (2015). Proteomics. Tissue-based map of the human proteome. Science.

[13] Wang J, Li J, Zhou L, Hou H, Zhang K (2024). Regulation of epidermal barrier function and pathogenesis of psoriasis by serine protease inhibitors. Front Immunol.

[14] Ayvaz HH, Öztürk KH, Seyirci MA, Atay E, Korkmaz S, Erturan İ (2023). The role of EREG, PTPN1, and SERPINB7 genes in the pathogenesis of psoriasis: may SERPINB7 be protective and a marker of severity for psoriasis?. Dermatol Pract Concept.

[15] Sakabe JI, Kabashima K, Sugita K, Tokura Y (2009). Possible involvement of T lymphocytes in the pathogenesis of Nagashima-type keratosis palmoplantaris. Clin Exp Dermatol.

[16] Li Y, Yu X, Pan C, Wang Y, Han J, Yao Z (2021). Effect of gentamicin ointment in patients with Nagashima-type palmoplantar keratosis: a double-blind vehicle-controlled study. Acta Derm Venereol.

[17] Brandt E, Harjama L, Elomaa O, Saarela J, Donner K, Lappalainen K (2024). A novel SERPINA12 variant and first European patients with diffuse palmoplantar keratoderma. J Eur Acad Dermatol Venereol.

[18] Zeber-Lubecka N, Suchta K, Kulecka M, Kluska A, Piątkowska M, Dabrowski MJ (2023). Exome sequencing to explore the possibility of predicting genetic susceptibility to the joint occurrence of polycystic ovary syndrome and Hashimoto’s thyroiditis. Front Immunol.

[19] Kubo A (2025). History and prospects of Nagashima-type palmoplantar keratosis, the most common palmoplantar keratoderma in East Asian populations. J Dermatol.

[20] Couto B, Neves C, Neves JS, Delgado L (2024). Thyroid function, autoimmunity, thyroid volume, and metabolic profile in people with Hashimoto thyroiditis. BMC Endocr Disord.

[21] Cui Z, Wang Z, Liu X, Cai Y, Xu X, Yang T (2019). Establishment of clinical diagnosis model of Graves’ disease and Hashimoto’s thyroiditis. J Transl Med.

[22] Zheng H, Gu L, Zhao F, Zhang C, Wang Z, Zhou H (2022). SerpinB7 deficiency contributes to development of psoriasis via calcium-mediated keratinocyte differentiation dysfunction. Cell Death Dis.

[23] Jensson BO, Arnadottir GA, Katrinardottir H, Fridriksdottir R, Helgason H, Oddsson A (2023). Actionable genotypes and their association with life span in Iceland. N Engl J Med.

[24] Wronska K, Halasa M, Szczuko M (2024). The role of the immune system in the course of Hashimoto’s thyroiditis: the current state of knowledge. Int J Mol Sci.

[25] Ralli M, Angeletti D, Fiore M, D’Aguanno V, Lambiase A, Artico M (2020). Hashimoto’s thyroiditis: an update on pathogenic mechanisms, diagnostic protocols, therapeutic strategies, and potential malignant transformation. Autoimmun Rev.

[26] Weetman AP (2021). An update on the pathogenesis of Hashimoto’s thyroiditis. J Endocrinol Invest.

[27] Petranovic OP, Gorges R, Giovanella L (2024). Autoimmune thyroid diseases. Semin Nucl Med.

[28] Steriade C, Bauer J, Bien CG (2025). Autoimmune encephalitis-associated epilepsy. Nat Rev Neurol.

[29] Yuan J, Qi S, Zhang X, Lai H, Li X, Xiaoheng C (2023). Local symptoms of Hashimoto’s thyroiditis: a systematic review. Front Endocrinol (Lausanne).

[30] Klubo-Gwiezdzinska J, Wartofsky L (2022). Hashimoto thyroiditis: an evidence-based guide to etiology, diagnosis and treatment. Pol Arch Intern Med.

[31] Hu Y, Feng W, Chen H, Shi H, Jiang L, Zheng X (2021). Effect of selenium on thyroid autoimmunity and regulatory T cells in patients with Hashimoto’s thyroiditis: a prospective randomized-controlled trial. Clin Transl Sci.

[32] Marenholz I, Grosche S, Kalb B, Rüschendorf F, Blümchen K, Schlags R (2017). Genome-wide association study identifies the SERPINB gene cluster as a susceptibility locus for food allergy. Nat Commun.

[33] Arnau-Soler A, Tremblay BL, Sun Y, Madore AM, Simard M, Kersten ETG (2025). Food allergy genetics and epigenetics: a review of genome-wide association studies. Allergy.

[34] Shin DH, Baek IC, Kim HJ, Choi EJ, Ahn M, Jung MH (2019). HLA alleles, especially amino-acid signatures of HLA-DPB1, might contribute to the molecular pathogenesis of early-onset autoimmune thyroid disease. PLoS One.

[35] Vargas-Uricoechea H (2023). Molecular mechanisms in autoimmune thyroid disease. Cells.

